# Endoscopic Stent Placement to Treat Gastric Leak Following Laparoscopic Sleeve Gastrectomy: the Bigger, the Better

**DOI:** 10.1007/s11695-022-05924-1

**Published:** 2022-01-28

**Authors:** Franck Billmann, Adrian Billeter, Anja Schaible, Beat Peter Müller-Stich

**Affiliations:** 1 Department of Surgery, University Hospital of Heidelberg, Im Neuenheimer Feld 420, 69120 Heidelberg, Germany; 2 Interdisciplinary Endoscopic Center, University Hospital of Heidelberg, Im Neuenheimer Feld 420, 69120 Heidelberg, Germany

To the Editor:

We would like to thank Sánchez-Luna et al. for their comments on our article “Endoscopic Stent Placement can successfully treat Gastric Leak following Laparoscopic Sleeve Gastrectomy If and Only If an Esophagoduodenal Megastent is used” [1].

While we certainly agree that the high efficacy of fully covered stents for post sleeve gastrectomy (SG) leak is related to its mechanism of action and especially by stenting a downstream stenosis [2], it is important to note that our study [1] focuses on acute/early gastric leaks (GL) after SG (interval from primary surgery to diagnosis of leak = 9.6 ± 7.3 days in the whole cohort). This is in stark contrast to most other studies that include both acute/early leaks and late/chronic fistulae.

Acute/early post-SG leaks are not only a serious but also a potentially life-threatening complication with a specific mortality ranging from 0.2 to 3.7% [3]. Mortality rises to 35% in patients who develop severe sepsis. Thus, rapid diagnosis and early management of the source of abdominal infection (i.e., gastric leak) are the cornerstones to prevent the sepsis cascade and progression to septic shock, further complications, and/or death [4–6]. In this setting, a success rate of 50–73% for conventional esophageal stent (CES) seems to us too risky. Furthermore, the authors argue that a 50% success rate is acceptable, but this is still much less than the > 90% success rate of our approach with the megastent (MS). In the elective bariatric-metabolic surgery setting, the goal is to improve long-term quality of life and to reduce long-term mortality by treating obesity and its related disease. The surgery itself, as well as the management of complications, should they arise, must therefore be as safe and effective as possible. Inadequate control at the source of infection has been shown to be one of the key prognostic factors of mortality in patients with intra-abdominal infections [7]. Hence, a 50–73% success rate in treating patients after metabolic-bariatric surgery is not sufficient in our view, especially when more effective therapies are available.

Furthermore, the investigations by Okazaki et al. and Hamid et al. [8, 9] should be interpreted very cautiously because these meta-analyses include studies with both acute/early leaks and chronic fistulae, limiting the generalizability of the data, since the literature consistently shows that acute/early leaks are very different from late/chronic fistulae. In fact, the authors’ flow chart of treatment selection clearly differentiates treatments based on the timing of the leak. Additionally, the meta-analysis by Okazaki et al. also included leaks after Roux-en-Y gastric bypass, which are very different from leaks after sleeve since they develop in a low-pressure system and do not have the same basis as sleeve leaks. Moreover, these meta-analyses are limited by the low quality of the included studies due to their small sample sizes, likely publication bias, and the overall heterogeneity of the patients included with acute/early leaks vs. late/chronic leaks and different procedure types.

Lastly, while stent migrations do occur at a relevant rate (30% in our cohort, which is in line with the published literature), they were without severe clinical consequences in our cohort and were repositioned endoscopically. Considering that alternative treatments such as EndoVac and others require regular endoscopic interventions, endoscopic stent repositioning in 30% of patients is an acceptable outcome, especially when the success rate of that therapy is so high (> 90%).

Finally, Sánchez-Luna et al. suggest that treatment of post-SG leaks should be individualized according to the clinical setting. We view the use of complex and individualized algorithms as a time-consuming approach that is not justified by the available evidence. In the case of acute/early leaks, rapid and effective control of the source of infection is pivotal to prevent a potentially fatal septic cascade. With this in mind, using simple, fast, and process-oriented algorithms (i.e., MS for all acute/early GL after SG) guarantees more efficient treatment decisions that achieve the best possible outcomes and ensure patient safety. The approach to late/chronic fistulae is more challenging and was not the aim of our study.

In conclusion, acute/early post-SG leaks require both effective and rapid treatment to prevent sudden deterioration. Due to their high efficiency, the simplicity of the therapeutic algorithm, and the speed of treatment implementation, MSs meet these requirements. We agree that late/chronic fistulae may require a different approach than acute/early leaks. However, as far as acute/early leaks are concerned, it can be concluded: the bigger, the better.
